# Citizens’ Perception on Air Quality in Portugal—How Concern Motivates Awareness

**DOI:** 10.3390/ijerph191912760

**Published:** 2022-10-05

**Authors:** Nuno Canha, Ana Rita Justino, Carla A. Gamelas, Susana Marta Almeida

**Affiliations:** 1 Centro de Ciências e Tecnologias Nucleares, Instituto Superior Técnico, Universidade de Lisboa, Estrada Nacional 10, Km 139.7, 2695-066 Bobadela, Portugal; 2 Instituto Politécnico de Setúbal, Escola Superior de Tecnologia de Setúbal, Centro de Investigação em Energia e Ambiente, IPS Campus, 2914-508 Setúbal, Portugal

**Keywords:** air pollution, air pollutants, sources, perception, air quality, citizens’ awareness, Portugal

## Abstract

This study aimed to understand the knowledge of Portuguese citizens about air quality and the extent to which the concerns about specific environmental problems can motivate their acquaintance of information. Moreover, this study also allowed to understand which information about air quality needs further dissemination to provide the citizens with all the available tools and the correct knowledge. For this, a national online survey about air quality perception was conducted, where 1131 answers were obtained and two different populations were compared: the general population and a sub-population from an urban-industrial area of Lisbon metropolitan area that had experienced frequent air pollution events in the past. Air pollution was considered the environmental topic of higher concern among this sub-population (61.4%), while in the general population it ranked thirdly (27.4%). Generally, the sub-population showed higher knowledge about air quality than the general population, with 61% being able to identify at least one air pollutant. The perception of the local air quality was also very different between populations, with 61% of the sub-population considering it poor or very poor, while only 14% of the general population had the same perception, which highlights the different levels of concern between populations. A weak knowledge about air pollutants (50% of the general population could not identify any air pollutant) and an erroneous perception of the contribution of the different pollution sources to air quality levels were found. More than 50% of the respondents of both populations were considered to not have enough information regarding the air quality in their area of residence, with the national air quality database being unknown to almost everyone. Overall, strong efforts should be made to increase the awareness about the importance of air quality, which may promote a higher acceptance of the implementation of future actions to improve air quality.

## 1. Introduction

Ambient air pollution has become a growing concern, mostly due to the rapid urbanization, industrialization, and traffic. In Europe, it is perceived as the second biggest concern (after climate change) and it is the most relevant environmental risk to human health [1]. Exposure to ambient air pollution is associated with a variety of health impacts such as cardiovascular diseases, cancer, respiratory diseases and mortality [2].

People’s understanding and response to ambient air pollution are vital to recognize the best mitigation measures concerning the protection of public health [3]. Thus, it is important to consider people’s perception and which factors may promote their behavioral changes, which may vary among groups and individuals [4]. The studies of air quality perception have not shown an association between the perceived air quality and the concentration of measured pollutants [5]. Instead, air quality perception seems to be influenced by sensory experience, awareness, and knowledge, the emotions it provides (such as nuisance), communication, and risk perception (which takes into account the psychological, social and cultural factors) [3,5]. Moreover, empowered populations with knowledge about air quality are typically more politically active to request actions from authorities to promote the control of air pollution.

For instance, a study developed in seven European countries (Austria, Belgium, Germany, Italy, Poland, Sweden and the United Kingdom) comparing the public perception of air pollution sources and the real-world situation through a survey involving 16,101 participants found that it existed a very high underestimation of the contribution of the agriculture sector to air pollution [6]. Moreover, this trend was common in all seven countries but demonstrated a small influence of gender, age, and socio-economic status of the respondents.

The present study aims to understand the awareness levels of Portuguese citizens regarding air quality along with their knowledge about this topic. Moreover, considering that some populations may be aware of the impact of local pollution sources in their daily life, this study also aimed to understand to which extension their concerns influence their awareness and knowledge about air quality. To achieve these goals, a national online questionnaire was made available during two months and the overall population was compared with a sub-population from an urban-industrial area of Portugal that is known to be aware of air pollution due to occasional settled dust events in the area [7,8].

## 2. Materials and Methods

### 2.1. Study Area

**Figure 1:**
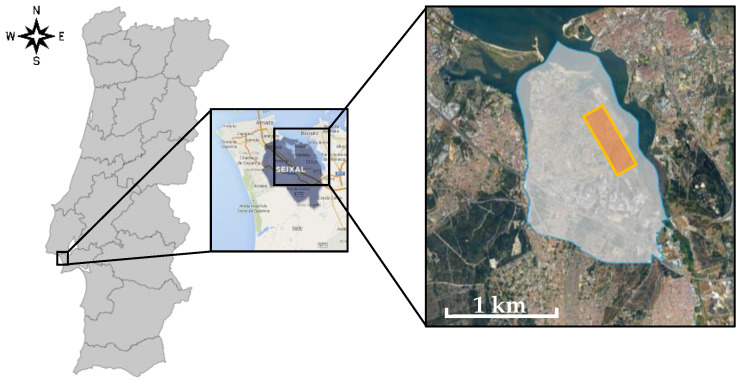
Location of the UFSAAPP area: (**left**) Framework of the study area (black rectangle) in Portugal mainland; (**center**) location of Seixal municipality highlighted; (**right**) location of UFSAAPP (whitish area with blue border represents the limits of the parish and orange represents the industrial area within it).

This study was conducted in Portugal and it compared the general Portuguese population and the population of a parish from a specific urban-industrial area where the inhabitants have presented several public complaints about the air quality (due to the operation of the industries located within its limits) [9]. This site is the parish of União das Freguesias do Seixal, Arrentela e Aldeia de Paio Pires (UFSAAPP), located in the municipality of Seixal (Portugal), which is integrated in the Metropolitan Area of Lisbon (Portugal), next to the Nature Reserve of the Tagus Estuary (Figure 1). With 167,294 inhabitants in 95.5 km^2^, the municipality of Seixal is one of the most densely populated municipalities in Portugal [10].

UFSAAPP is considered a typical urban-industrial area that comprises a densely populated residential area and a significant network of highways and national roads (EN10, A2-IP7 and A33), along with an industrial park where the facilities of different small and medium-sized industries are located (e.g., steelworks, lime factory and metal waste management and treatment [11,12,13]).

### 2.2. Questionnaires

A questionnaire was created to assess the perception of the Portuguese population regarding air quality. The questionnaire had five different sections: (i) participants’ demographic characteristics; (ii) perception towards air quality, including knowledge about air pollutants and their sources; (iii) perception of the impacts of air quality, (iv) sources of information, and (v) knowledge of applicable regulations for controlling air quality. Overall, the questionnaire had a total of 36 questions and it was created based in the questionnaires developed by the project RISKAR LX [14]. The questionnaire is available in the Supplementary Materials (English version).

The questionnaire was disseminated by social media and an invitation was sent to all Portuguese municipalities to share it with their citizens. The questionnaire was available online from 1 February to 26 June 2020 for anonymous answer and a total of 1131 answers were gathered. Only participants with 18 or more years old were considered.

The survey implementation and data handling were conducted according to the guidelines of the Declaration of Helsinki. The online questionnaires were presented to the participants alongside a short introductory summary, which defined the objectives of the study and ensured the anonymity and confidentiality of the provided information. For data analysis, all answers were codified and treated anonymously.

### 2.3. Statistical Analysis

Statistical tests were carried out using the supplement XLSTAT of Microsoft Excel (Addinsoft, Paris, France). Chi-square tests of independence were used to examine if participants’ gender, age, educational level, monthly income, among others, were associated with the participant’s level of concern about air quality and the evaluation of the air quality (with a significance level of 0.050).

## 3. Results and Discussion

### 3.1. Characteristics of Respondents

Table 1 presents the answer rate obtained by Portuguese district, where answers from all the Portuguese districts were obtained. Excluding the answers from the parish of UFSAAPP, the distribution among districts is more even, despite a great contribution from Lisbon district (37.3% instead of only 22.0%) and a lower contribution from Porto district (3.8% instead of 17.3%).

Table 2 provides the general demographic information of the respondents of the survey for both populations. For the general population, which included 1004 respondents, 61.9% were female and 37.7% were male, with the age groups of 21–25 and 41–45 years old as the ones with a higher percentage of respondents, namely, 17.6% and 17.2%, respectively. The most common school level was degree (47.9%), followed by high school (29.2%), while the most prevalent working status was active (78.3%) and the most common monthly income was between 1001–2000€ (35.8%). The demographic information of UFSAAPP population has a similar structure (despite not being statistical equal) and gathered a total of 127 respondents. Regarding the gender balance of this population, 63.0 % were female and 34.6% were male, with the age groups of 41–45 and above 60 years old being more represented (with 26.0 and 14.2%, respectively). Similar to the general population, the UFSAAPP population was mainly characterized by respondents with degree (44.9%) or high school (42.5%), being active regarding their working status (77.2%), with a monthly income of between 1001 and 2000€ (40.2%).

As described in the methodology, this survey gathered information regarding five different topics and the results were evaluated comparing the general population (*n* = 1004, all individuals that answered the survey, except the individuals from the parish of UFSAAPP) and the population of UFSAAPP (*n* = 127), the study area that is urban-industrial and whose population is known to be aware about air pollution due to occasional settled dust events in the area [9].

### 3.2. Issues of Concern

**Figure 2:**
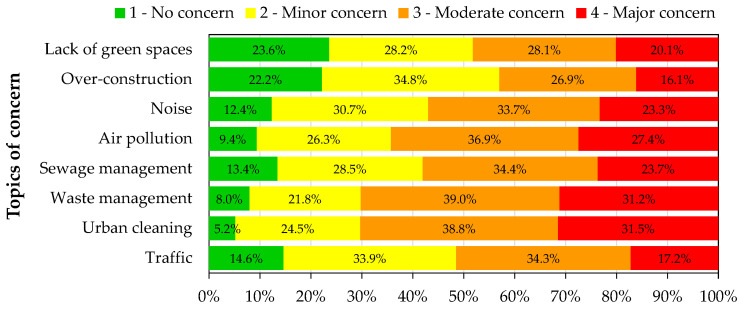
Level of concern of the general population regarding different environmental topics.

**Figure 3:**
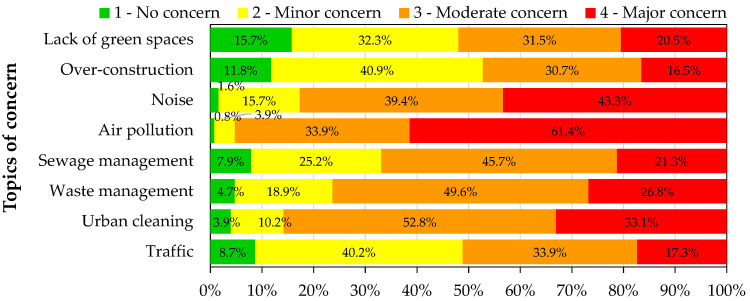
Level of concern of the UFSAAPP population regarding different environmental topics.

The main issues of concern of the two populations were assessed to understand how the air quality is perceived as an important issue within different topics. For that, the participants were asked to rate from 1 (no concern) to 4 (major concern) different environmental topics that could potentially affect their life. Figure 2 and Figure 3 present the percentages of the level of concern for the different environmental topics for the general and UFSAAPP populations, respectively.

For the general population, the environmental topics that gather the highest levels of concern are urban cleaning (31.5%) and waste management (31.2%), followed in third position by air pollution (27.4%). This rank changes greatly when focusing on the UFSAAPP population, where air pollution gathers the highest level of concern (61.4%), followed by noise (43.3%) and then, urban cleaning (33.1%).

The association of the top-two main concern issues revealed by UFSAAPP population (air pollution and noise) was already identified previously [15] and usually it is difficult to distinguish them since they have common sources [16], such as transports, industry, agriculture and, with minimal contribution, household and neighborhood. Similar trend was already verified elsewhere [17], with the noise annoyance felt by citizens being directly related with their perception of a worst air quality.

The potential association between the level of concern regarding air quality and sociodemographic characteristics was assessed by χ^2^—test. In the general population, the level of concern was found dependent on the education level (χ^2^ = 37.6, *p*-value = 0.004) and the district of residence (χ^2^ = 138.7, *p*-value < 0.0001). Individuals with the basic school (six years) presented a higher level of concern regarding air quality (considering the categories “moderate concern” and “major concern” together, totalizing 91% of the individuals with the basic school) than the remaining individuals with different scholar levels (with an average of 66% for the same categories). However, it is important to highlight that the population with basic school only represents 1% of the total participants. In fact, the educational level is an important factor and, for instance, improving the level of education per capita has been shown to promote the gradual decrease of the impact of increased air pollution on public health damage [18].

The district of residence was also a factor of influence on the concern level, since the individuals that lived in districts in the country inland or islands (which are districts of low population density) presented a lower level of concern than the ones that lived in districts with higher population density (such as Braga, Guarda, Lisbon, and Setúbal), whereas the individuals of Setúbal presented the highest level of concern among all (with 83% of the respondents from Setúbal reporting “moderate concern” and “major concern”).

### 3.3. Perception of Air Quality

**Figure 4:**
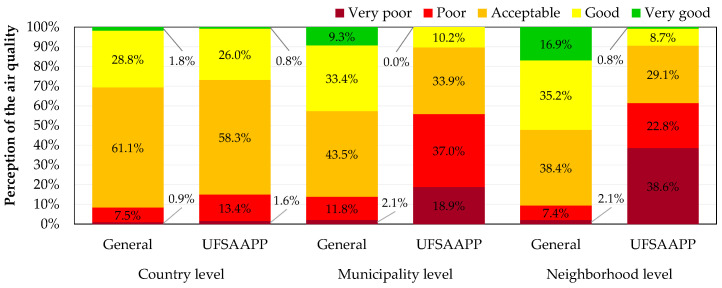
Assessment of the perception on air quality in Portugal, at country, municipality and neighborhood levels for the general and UFSAAPP populations.

Figure 4 provides the perception of state of the air quality by the citizens in different levels (country, municipality and neighborhood), considering the two types of study populations: general and UFSAAPP. As described above, the general population considers the citizens that reside in all districts of the country, except in the UFSAAPP area, while the UFSAAPP population refers exclusively to the inhabitants that reside UFSAAPP parish.

At a country level, both populations have a similar perception of the air quality of the country, with “acceptable” air quality being the most common perception (61.1 % for the general population and 58.3% for the UFSAAPP population), followed by “good” air quality (28.8% for the general population and 26.0% for the UFSAAPP population) and then, by “poor” air quality (7.5% for the general population and 13.4% for the UFSAAPP population). The perception of good or very good air quality is similar between populations: 30.6% and 26.8% for the general and UFSAAPP populations, respectively. However, when assessing the air quality of the municipality or the neighborhood, the perceptions differ greatly between populations, with the UFSAAPP population clearly having a worst perception. Both at municipality and neighborhood levels, 55.9% and 61.4% of the UFSAAPP respondents have a “very poor” or “poor” perception of the air quality, respectively. At the municipality level, 13.9% of the general population have the perception of “poor” or “very poor” air quality, while this perception decreases to 9.5% when focusing on the neighborhood level.

This great difference on the perception of the air quality between the general and UFSAAPP populations at the local level highlights the concerns of the UFSAAPP population, reflected by the high level of awareness and sensibility of the UFSAAPP population toward this topic [9].

### 3.4. Identification of Pollution Sources

**Figure 5:**
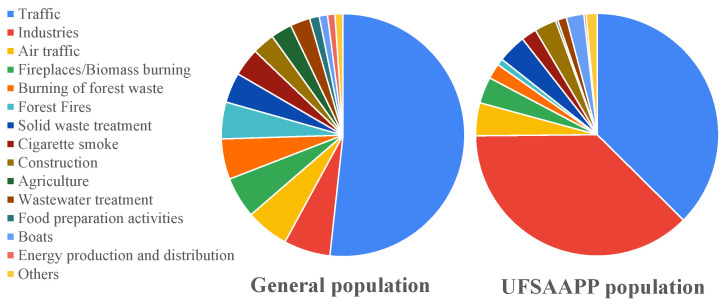
Air pollution sources identified by the general and the UFSAAPP populations.

The survey also aimed to identify the knowledge of the participants regarding pollution sources and air pollutants. Figure 5 provides the main air pollution sources identified by the general and UFSAAPP populations in their area of residence.

For the general population, traffic was the most frequently identified pollution source, being referred by 51.7% of the respondents in the general population, which agrees with other studies where traffic was highlighted as one of the main sources of air pollution: China (with 78.5%) [19], Malaysia (where “motor vehicle emissions” were ranked as the most significant contributor to air pollution in their residential areas) [20], Mexico (with 50% highlighting cars and trucks) [21], and in seven European countries (ranging from 29.1% in Germany to 42.4% in Sweden) [6]. Industry (6.2%) and air traffic (5.7%) were considered as the second and third main pollution sources in our study. However, it is relevant to highlight that the study developed in seven European countries [6] (Austria, Belgium, Germany, Italy, Poland, Sweden, and United Kingdom) showed industry as the main contributor to the air pollution, while traffic was identified only as the second main pollution source. In the present study, it was possible to identify a pattern where the participants from districts of the country inland indicated pollution sources more associated with the rural areas, such as the burning of green wastes, forest fires, and fireplaces/biomass burning (for home heating).

The main air pollution sources perceived by the UFSAAPP population were traffic and industries (both with 37.4%), followed in third by air traffic (4.4%). In the UFSAAPP population, industries are considered one of the two main sources of air pollution (six times more than in the general population), probably due to the fact that UFSAAPP has suffered from several punctual pollution events (such as events of deposition of coarse particles in the area [7,9]), which have been attributed to the local industries [8] by the population. The proximity to an industrial area may affect the individual perception [22], which can be confirmed by the higher levels of concern of the UFSAAPP population since the heavy industry park is at walking distance from the residential area of UFSAAPP, along with the existence of many small and medium sized industries dispersed by the area. Another relevant issue is that, typically, the individual control of pollution sources from industries is minimum, which results in a higher degree of attention to those sources, including an increase of exposure reports from the population [23]. Therefore, taking into account that the UFSAAPP population has previous concerns and complaints regarding the local industries, it would be expected that industries would be considered by them one of the main pollution sources.

**Figure 6:**
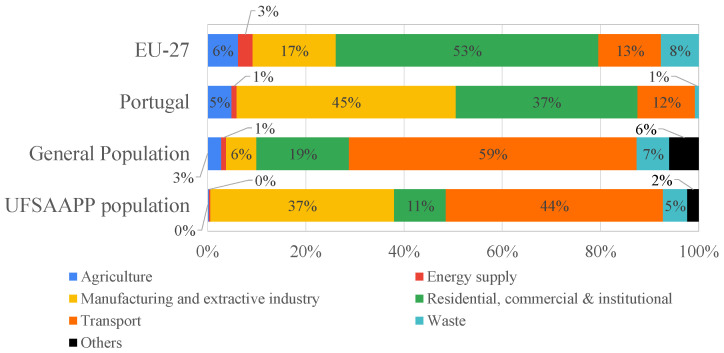
Emission sources of PM_2.5_ in 2019 for EU-27 and Portugal [24], and the main air pollution sources perceived by the general and the UFSAAPP populations in the present study.

However, considering PM_2.5_ as a proxy for air pollution, since it is the air pollutant responsible for more premature deaths in EU countries than any other pollutants (for instance, a total of 4900 premature deaths were attributable to PM_2.5_ exposure in Portugal in 2019 [24]), the real direct emission sources for PM_2.5_ may differ from the citizens’ perception. Figure 6 presents the emission sources of PM_2.5_ in 2019 for EU-27 and Portugal, along with the main air pollution sources identified by the general and the UFSAAPP populations (using a categorization of only seven pollution sources, where, for instance, traffic, air traffic and boat were framed in a single category called “transport”).

The major differences between the official and real-world data regarding the PM_2.5_ emissions sources (both from EU-27 and Portugal) and the sources identified by the two studied populations are:

(i) The overestimation of the contribution of transport sector to air pollution by the public perception (59% and 44% for the general and UFSAAPP populations, respectively, against 12% of real contribution of transports to PM_2.5_ levels in Portugal);
(ii) The underestimation of the “residential, commercial and institutional” source by the public perception (19% and 11% for the general and UFSAAPP populations, respectively, against 37% of the contribution of this source to the PM_2.5_ levels in Portugal)
(iii) The great underestimation of the “manufacturing and extractive industry” contribution by the general population (6%) regarding the real contribution of 45% in Portugal (where the average contribution in the EU-27 is 17%). The UFSAAPP population indicated a value of 37% (probably, as described previously, due to their own concerns regarding the local industries), which is close to the real contribution verified in Portugal. However, it is relevant to highlight that the real contribution is higher than the perception of both populations, which indicates that the common citizen is not aware of the impact of industry in the air quality.
(iv) The negligible contribution of agriculture to air pollution, perceived by the general (3%) and the UFSAAPP (0%) populations, while the real world data indicate a higher contribution of 5% in Portugal and of 6% in the EU-27. However, if considering the secondary PM sources, such as SO_2_ and NO_X_ from the industry contribution and NO_X_ from traffic emissions, combined with ammonia emission (from the agricultural sector), the solo contribution of agriculture may be very significant to air pollution levels and, typically, it is neglected by the public perception [6].

### 3.5. Identification of Air Pollutants

The citizens’ knowledge about air pollutants was also assessed in this survey, where the participants indicated which air pollutants they knew about (open question). For the identification of air pollutants and summarizing the results, the main air pollutants were considered individually (namely, CH_4_, CO, CO_2_, NO_X_, O_3_, PM, SO_X_), while other identified air pollutants were considered as “Others”. When a respondent did not supply any answer or answered “I do not know”, it was quantified as “DK/NO”. Answers that considered pollution sources (e.g., traffic) instead of air pollutants were not considered. For the category “PM”, all answers related with particulate matter were considered (such as “particles”, “dust”, “aerosols”, “black carbon”, “PM_2.5_”, and “PM_10_”, among others).

**Figure 7:**
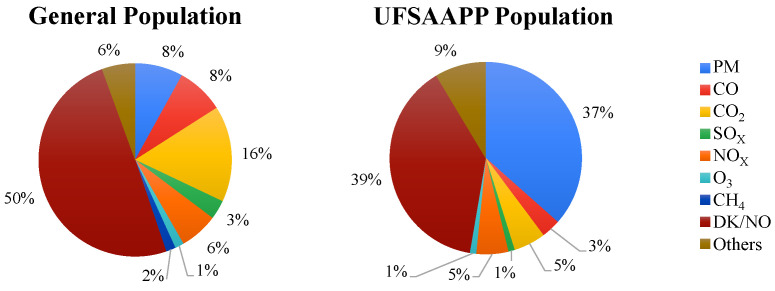
Air pollutants identified by the two studied populations. DK/NO stands for “Do not know/No answer”.

Figure 7 provides the air pollutants identified by both populations. Half of the general population (50%) indicated that they could not identify any air pollutant, while only 39% of the UFSAAPP population provided the same answer. The higher knowledge about air pollutants in the UFSAAPP population is probably due to their higher sensibility and awareness regarding the topic of air pollution, as described previously, since that population has experienced several air pollution events in the past, which promoted their need to acquire knowledge about air pollutants.

For the general population, the air pollutant most frequently identified was CO_2_ (16%), followed by PM and CO (both with 8%). Carbon dioxide is typically known by the citizens since it is one of the most discussed air pollutants in the media (mainly television), especially due to its association with climate change and all the global efforts that are being conducted to carbon neutrality [25]. The air pollutant more identified by the UFSAAPP population was particulate matter (37%), followed by “Others” (9%) and, in third, CO_2_ and NO_X_ (both with 5%). In the UFSAAPP population, settled dust events are one of the main problems affecting the population (which they assume to be related to the local industries) and, therefore, it seems natural that the air pollutant that most respondents are familiar to is PM. Moreover, it is relevant to highlight that the concerns of the UFSAAPP population represent probably one of the main triggers for their environmental awareness regarding air pollutants.

### 3.6. Information about Air Quality

**Figure 8:**
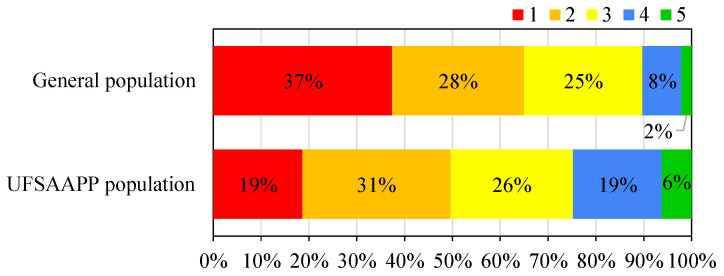
Level of how much citizens feel informed about air quality in their area of residence, ranging from 1 (“not at all”) to 5 (“very much”).

Information about air quality and, consequently, about air quality indexes, has to be reliable and understandable to all the population, since it is through trustworthy information that it is possible to increase the awareness of the general public regarding this issue. In order to understand if the citizens feel that they are informed about the local air quality, all participants were inquired about “Do you feel informed about the air quality in your area of residence?” and asked to answer, ranging from “Not at all” to “Very much”. Figure 8 provides the results for both populations.

The majority of the general population (65%) feels that they do not have enough information about air quality in their area of residence (considering the grades “1” and “2”), with only 10% answering that they felt well informed about it (considering the grades “4” and “5”). Half of the UFSAAPP population (50%) also considers not to have sufficient information about air quality (considering the grades “1” and “2”), but an increase of the informed population is observed with 25% stating that they felt well informed about air quality in their local area (considering the grades “4” and “5”). The feeling of insufficient information about air quality (considering grades “1” and “2”) in the present study is slightly lower than the expressed by Portuguese citizens in a European survey carried out on September 2019 [26], where a mean of 54% of the respondents from 28 member states of European Union considered that they did not feel well-informed about air quality problems in their country, ranging from 75% in Portugal to 18% in Finland. This survey concluded that only 25% of the Portuguese population considered that was well-informed about air quality problems in their country, a similar result to the one obtained for the UFSAAPP population.

**Figure 9:**
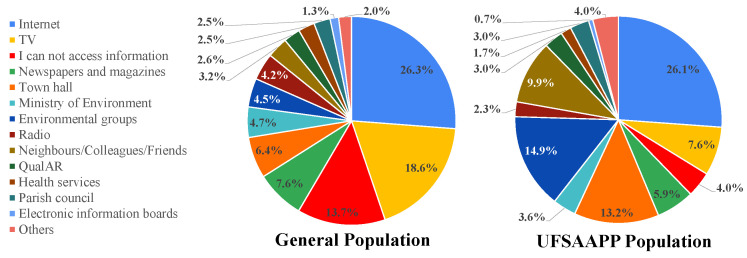
Sources of information about air quality for general and UFSAAPP populations.

Figure 9 provides the main sources of information of both studied populations. In the general population, 13.7% of the participants stated that they have difficulties accessing information about the local air quality, while only 4.0% of the UFSAAPP population reported the same problem. For both populations, the main source of information is the internet with around 26%. For the general population, the second main source of information is TV (18.6%), followed by “Newspapers and magazines” (7.6%). For the UFSAAPP population, the second main source of information is “Environmental groups” (14.9%), followed by “Town hall” (13.2%).

A study conducted in China concluded that the main sources of information regarding air pollution were TV/radio (20.8%), Internet (18.9%) and Newspapers/magazines (18.7%) [27], a similar trend to the one found in the present study for the general population. In a study developed in the United Kingdom [25], the main sources of information about air quality that were identified by the participants were internet (44.7%), local council (29.3%), and the media services, namely, radio/TV/newspapers (13.2%). The main difference registered in our study is the contribution of “Environmental groups” as information source for the UFSAAPP population. However, this may be due to the very specific characteristics of this population since they have experienced several air quality problems in the past and, therefore, they seek information on the environmental groups that target their issue (both locally and nationally), along with the local Town hall. Some of the environmental groups were created by citizens specifically due to the air pollution events that occurred in the UFSAAPP area, and others are environmental non-governmental organizations of national range [28]. Once again, this highlights that the concern regarding local air quality problems potentiates the citizens to seek information and, typically, using a more local approach.

However, these results also show that the Portuguese governmental online database of air quality (created by the Portuguese Environment Agency), which is of free access and regularly updated, called “QualAR” (https://qualar.apambiente.pt/ accessed on 17 July 2022) is not a common source of information for the citizens. Only 2.6% and 3.0% of the participants from the general and the UFSAAPP populations, respectively, identified QualAR as a source of information. This fact highlights the need for the governmental stakeholders to promote the awareness of this tool, to empower the general public with knowledge about their local air quality.

### 3.7. Impacts of Air Quality in the Daily Life

**Figure 10:**
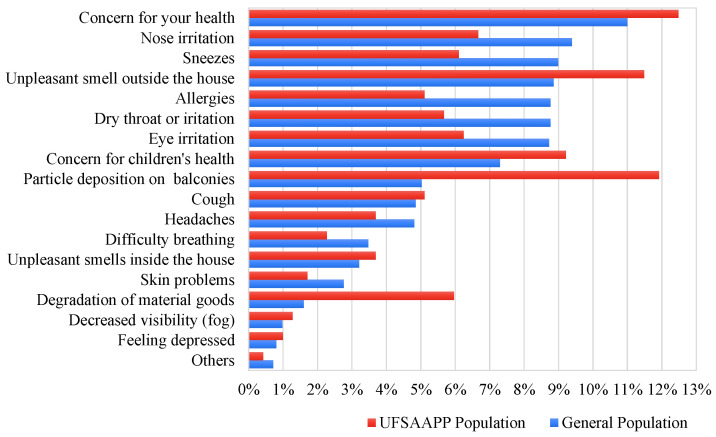
Perception of the impacts of air quality problems on the general and UFSAAPP populations.

Both populations were asked if they felt that, at some moment, they were already affected by air quality. In the general population, 52.9% revealed that they had already felt affected by air quality, while in the UFSAAPP population this percentage reached 86.8%, which highlights the strong feeling of the UFSAAPP population that their residence area is affected by local air quality problems. Figure 10 presents the incidence of the main impacts of air quality problems perceived by the respondents.

Both populations considered that air quality problems had an impact on their own health, with 11.0% of the general population and 12.5% of the UFSAAPP population reporting this. For the general population, the main issues of impact of air quality problems were health-related issues, such as nose irritation (9.4%) and sneezes (9.0%). The UFSAAPP population focused on the remaining main impacts of air quality problems, such as on events of particle deposition on balconies (11.9%) and unpleasant smell outside the house (11.5%), which are directly related to the previous complaints by this population, as already reported above. Additionally, in this population, the concern regarding the deterioration of material goods due to air pollution was also very high (6.0%), when comparing with the general population (1.6%).

**Figure 11:**
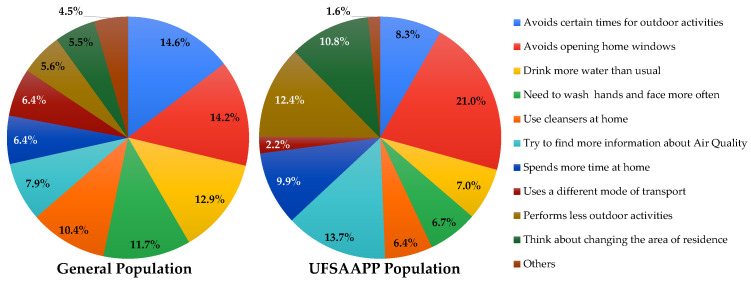
Changes made in the daily life when citizens felt affected by air pollution, for the general population (**left**) and the UFSAAPP population (**right**).

When asked if the participants have made changes in their daily life when they felt that were being affected by air pollution, only 31% of the respondents of the general population answered affirmatively, while 69% of the UFSAAPP population reported the same behavior. Figure 11 describes the main changes made by citizens in their daily life when they felt being affected by air pollution.

For the general population, the main changes were to avoid certain time periods for outdoor activities (14.6%), to avoid opening home windows (14.2%), and to drink more water than usual (12.9%). For the UFSAAPP population, the main changes were to avoid opening the home windows (21.0%), to try to find more information about air quality (13.7%) and to perform less outdoor activities (12.4%). It is relevant to highlight that 10.8% of the respondents from the UFSAAPP population revealed considering changing their area of residence due to air pollution issues, which is the almost the double verified in the general population (5.5%). This issue reveals that air quality may be a relevant issue to consider in the real estate market.

### 3.8. To Which Mitigation Measures to Improve Air Quality Are the Citizens More Favorable to?

**Figure 12:**
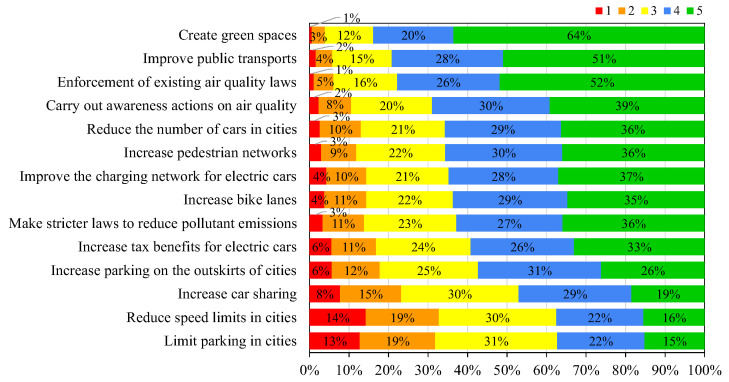
Degree of priority given by the citizens of the general population to different measures to improve air quality (ranging from 1 for “minimum priority” to 5 for “maximum priority”).

**Figure 13:**
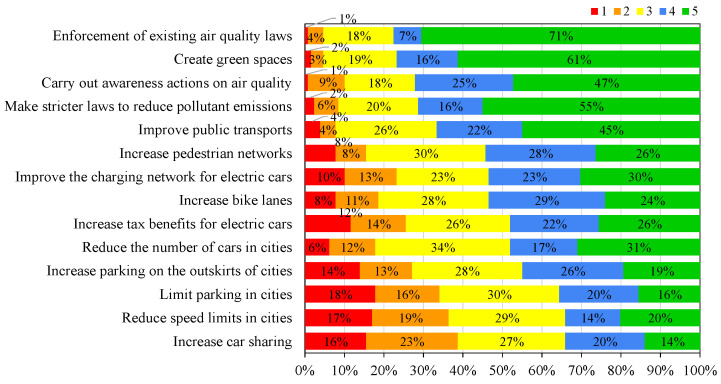
Degree of priority given by the citizens of the UFSAAPP population to different measures to improve air quality (ranging from 1 for “minimum priority” to 5 for “maximum priority”).

The study conducted by the EU identified that 67% of the Portuguese population considered that the public authorities were not doing enough to promote good air quality to their citizens [26]. To understand which mitigation measures the citizens are more receptive to adopt or to support, all participants were asked the degree of priority (ranging from “1” as minimum priority to “5” as maximum priority) that they attributed to different measures to improve air quality. Figure 12 and Figure 13 present the results obtained for the general and UFSAAPP populations, respectively.

The general population considered that the measures with higher priority (classifications of “4” and “5”) were to create green spaces (84%), to improve public transports (79%), and to enforce the existing air quality laws (78%). The UFSAAPP population considered that the measures with higher priority were the enforcement of existing air quality laws (78%), the creation of green spaces (77%) and to carry out awareness actions on air quality (72%). The difference on the priority rank of the mitigation measures considered by the UFSAAPP may be influenced again by their own experience of previous episodes of air pollution events and also by living in an industrial-urban area, which makes them more favorable to the enforcement of existing air quality legislation, in terms of compliance of the industry emissions with their environmental licenses and legislated limit values. Comparing with the European study, the EU-28 citizens (including the Portuguese participants) considered that the most effective way to tackle the air quality problems would be to apply stricter pollution controls on industrial and energy-production activities (44% of the EU-28 participants and 43% of the Portuguese participants) [26].

### 3.9. Considerations

Although the survey conducted in this study gathered answers from all the Portuguese districts, it is important to highlight that the adequate percentage of representativeness among regions was not achieved for the general population of Portugal. Moreover, the stratification of the characteristics between the studied populations are not equal, which may influence the analysis. The gender variability of participants is also not representative of the Portuguese population (52% Female and 48% Male) [10], with the present study gathering around 62–63% of answers from females. The influence of gender on air quality perception is known with females generally perceiving more air pollution [29]. However, the present results still provide new and valuable insights regarding the perception of the citizens on air quality issues, which may be determinant on the approaches chosen by public authorities to maximize their dissemination strategies targeting the citizens. The comparison with a specific population that is more aware of air pollution issues (which is reflected by their higher level of knowledge regarding the air pollution issues) highlights that real life concerns potentiate the search for information and empowerment of the citizens regarding this topic (which is the case of the specific population since they have been subject to occasional air pollution events, which motivated their search for knowledge to understand the potential implications of those events). Some main ideas are important to retain and to highlight, such as:

- Great differences were found when comparing both studied populations. It was found that UFSAAPP population showed a higher concern regarding air pollution in comparison with the general population (with 61.4% of the UFSAAPP population considering it the main environmental concern). This higher concern was also demonstrated by their significant knowledge of possible pollutants and higher need to search for information about the topic. Furthermore, the UFSAAPP population considered industry as the main source of air pollution (with 37.4% of the answers) along with traffic (37.4%). This trend was not found in the general population, where traffic was appointed as the main pollution source (51.7%), followed by the industry (only with 6.2%). This fact highlights the concern and awareness that the UFSAAPP population has regarding industry as a pollution source.
- A great part of the Portuguese population feels that are not suitably informed regarding the air quality levels in their area (65%), with only 10% stating that they feel well informed about it. It would be important that reliable and easily understandable information about air quality could be of easy access and widespread throughout the country; this would empower the citizens regarding air quality and promote their future engagement in mitigation actions to improve air quality. This is a crucial area that policy makers and governmental bodies should focus on in order to decrease the national environmental illiteracy regarding this topic and promote behavioral actions in the population that can lead to an improvement in the air quality.
- Unfortunately, this study revealed that the governmental online and free database of information about local air quality (QualAR) is almost unknown by the Portuguese population (being acknowledged to be a source of information by only 3% of the participants). This highlights that the current dissemination strategies are not working or are not enough to reach the general public, which should be targeted to maximize all the potentialities of this available tool.
- Due to the citizens’ awareness about air pollution and its health and daily life impacts, air quality is a relevant issue in the real estate market (10.8% of citizens consider to change their area of residence when under air pollution events). This fact potentiates the engagement of local governmental authorities to implement measures to improve local air quality, to improve the quality of life of their citizens, to attract new inhabitants to the area and to improve the touristic potential of their municipalities.

A follow-up study of this survey should be conducted in the future targeting to achieve a higher participation (to obtain a higher sample number and representativeness of the Portuguese population), in order to evaluate the changes on the perception of citizens, which will provide insights regarding whether the current strategies of awareness regarding air pollution are effective or not. Moreover, it would also be important to allow the participants to provide their opinions on some specific issues, such as their best suggestions/strategies to improve air quality.

It is important also to highlight that strategies to promote a higher public acceptance of mitigation policies rely on actions with the population to promote their empowerment and engagement, such as health literacy programs, awareness raising campaigns, and public participation activities [30].

## 4. Conclusions

This study allowed to assess the perception of the air quality by the population of an area affected by air pollution events and to compare it with the general population. This sub-population showed to have a higher level of knowledge and awareness regarding the topic of air pollution, considering it as the main environmental concern (while the general population ranked it only in third). Moreover, the sub-population also showed more knowledge about air pollutants than the general population, with 61% being able to identify at least one air pollutant, while half of the general population did not manage to identify any air pollutant. The perception of the local air quality (at the neighborhood level) was also very different between populations, with 61.4% of the sub-population considering it poor or very poor, while only 9.5% of the general population had the same perception, which highlights the different levels of concern between populations.

Some issues related to air quality still demonstrate a weak knowledge among the studied populations, namely the identification of air pollutants (50% of the general population could not identify any air pollutant) and an erroneous perception of the contribution of the different pollution sources to air quality levels (traffic was identified as the main source by both populations, with an overestimation of its impact between four and five times higher than the real emissions impact on air quality levels).

Despite both populations having some knowledge about air quality, still more than 50% of both populations feel that they do not have enough information regarding the air quality in their area of residence. The current tools of air quality information made available by governmental bodies are unknown to most of the population. Therefore, it is important and crucial to invest in the empowerment of the populations regarding environmental knowledge (especially air quality), since it is a strategy to increase and obtain their receptivity to the implementation of future actions to improve air quality.

The main actions considered by both populations to improve air quality to which the citizens are more receptive to include enforcement of existing air quality laws, creation of green spaces, implementation of awareness actions on air quality, and improvement of public transportations.

Overall, our study highlights several weaknesses regarding the citizens’ knowledge about air quality and the need for the governmental stakeholders to promote the awareness of the importance of air quality, including which tools are available regarding information and monitoring.

## Figures and Tables

**Table 1:** Portuguese population per district and answer rate to the survey per Portuguese district, considering all the answers received and excluding the ones from UFSAAPP parish (*n* = 129).

	Population		All Answers to Survey		Answers to Survey, Excluding from Seixal Municipality	
District	Inhabitants	%	*n*	%	*n*	%
Aveiro	700,964	6.8	16	1.4	16	1.6
Beja	144,410	1.4	4	0.4	4	0.4
Braga	846,515	8.2	61	5.4	61	6.1
Bragança	122,833	1.2	11	1.0	11	1.1
Castelo Branco	177,912	1.7	32	2.8	32	3.2
Coimbra	408,631	3.9	10	0.9	10	1.0
Évora	152,436	1.5	5	0.4	5	0.5
Faro	467,495	4.5	97	8.6	97	9.7
Guarda	143,019	1.4	5	0.4	5	0.5
Leiria	458,679	4.4	109	9.6	109	10.9
Lisboa	2,275,591	22.0	377	33.0	374	37.3
Portalegre	104,989	1.0	11	1.0	11	1.1
Porto	1,786,656	17.3	38	3.4	38	3.8
Região Autónoma da Madeira	251,060	2.4	2	0.2	2	0.2
Região Autónoma dos Açores	236,657	2.3	5	0.4	5	0.5
Santarém	425,431	4.1	32	2.9	33	3.3
Setúbal	875,656	8.5	275	24.7	148	14.8
Viana do Castelo	231,488	2.2	3	0.3	3	0.3
Vila Real	185,878	1.8	5	0.4	5	0.5
Viseu	351,592	3.4	33	2.9	33	3.3
Total	10,347,892	100.0	1131	100.0	1002	100.0

**Table 2:** Sociodemographic characterisation of the respondents, where *n* is the number of individuals in each category.

		Population			
		General		UFSAAPP	
Characteristic	Category	*n*	%	*n*	%
Gender	Female	621	61.9	80	63.0
	Male	379	37.7	44	34.6
	Prefer not to answer	4	0.4	3	2.4
Age	<20	40	4.0	2	1.6
	21–25	177	17.6	7	5.5
	26–30	60	6.0	2	1.6
	31–35	62	6.2	13	10.2
	36–40	100	10.0	9	7.1
	41–45	173	17.2	33	26.0
	46–50	142	14.1	17	13.4
	51–55	98	9.8	12	9.4
	56–60	92	9.2	14	11.0
	>60	60	6.0	18	14.2
School Level	Primary School (4 years)	5	0.5	1	0.8
	Basic school (6 years)	11	1.1	0	0.0
	Middle school (9 years)	31	3.1	3	2.4
	High school (12 years)	293	29.2	54	42.5
	Degree	481	47.9	57	44.9
	Master	159	15.8	12	9.4
	PhD	24	2.4	0	0.0
Working Status	Student	152	15.1	6	4.7
	Active	786	78.3	98	77.2
	Retired	32	3.2	17	13.4
	Unemployed	25	2.5	4	3.1
	Others	9	0.9	2	1.6
Monthly income	<300€	17	1.7	0	0.0
	301–635 €	66	6.6	8	6.3
	636–900 €	186	18.5	18	14.2
	901–1000 €	132	13.1	11	8.7
	1001–2000 €	359	35.8	51	40.2
	2001–3000 €	54	5.4	11	8.7
	>3000 €	14	1.4	0	0.0
	Not applicable	120	12.0	8	6.3
	Prefer not to answer	56	5.6	20	15.7
	Total	1004	100.0	127	100.0

## Supplementary Materials

The following supporting information can be downloaded at: https://www.mdpi.com/article/10.3390/ijerph191912760/s1, File S1: Questionnaire on the perception of air quality in Portugal.
