# Supplementary material for: Citizens’ Perception on Air Quality in Portugal—How Concern Motivates Awareness
Source: Int J Environ Res Public Health. 2022 Oct 5;19(19):12760. doi: 10.3390/ijerph191912760 (PMC9566417; doi:10.3390/ijerph191912760)
Supplement: Supplementary file 1 [file ijerph-19-12760-s001.zip › ijerph-1846908-supplementary.pdf]

Supplementary Information

Questionnaire on the perception of air quality in Portugal

(translation to English)

* mandatory answer

7.1. Participant characterisation

I. District of residence:*

○ Aveiro	○ Lisboa
○ Beja	○ Portalegre
○ Braga	○ Porto
○ Bragança	○ Santarém
○ Castelo Branco	○ Setúbal
○ Coimbra	○ Viana do Castelo
○ Évora	○ Vila Real
○ Faro	○ Viseu
○ Guarda	○ Autonomous Region of Madeira
○ Leiria	○ Autonomous Region of Azores

II. Municipality of residence:* ______________________________

III. Parish of residence:* ______________________________

IV. Postal code of residence:* ______________________________

V. Type of housing:*

○ Detached house ○ Apartment

VI. If it is an apartment, please indicate which floor:

○ Ground floor	○ 1st floor
○ 2nd floor	○ 3rd floor
○ Other: ____________________	

VII. Your house is located in an area with:*

	1	2	3	4	5	
No traffic	○	○	○	○	○	A lot of traffic

VIII. Age:* ______________________

IX. Gender:*

- Female
- Male
- Prefer not to disclosure

X. Highest level of education completed:*

- Uneducated
- 1st Cycle of Basic Education
- 2nd Cycle of Basic Education
- 3rd Cycle of Basic Education
- High School
- Degree
- Master
- Doctorate

XI. Work status:*

- Active
- Retired
- Unemployed
- Student
- Home tasks
- Other: __________________

XII. Current profession or last practiced:*

____

XIII. On average, what is your monthly salary?*

- Less than 300€
- 300-635€
- 636-900€
- 901-1000€
- 1001-2000€
- 2001-3000€
- Not applicable
- I prefer not to answer

XIV. Number of people in the household (counting you):* _____________

XV. Number of people in the household with less than 12 years old:* ___________

XVI. Number of people in the household aged between 12 and 18 years old:*_______

XVII. Do you smoke?*

- Yes
- No

XVIII. Select the diseases/health problems that you have:*

- Eczema
- Asthma

- ☐ Migraine
- ☐ Allergy
- ☐ Cholesterol
- ☐ Arterial hypertension
- ☐ Diabetes
- ☐ Depression
- ☐ Anxiety
- ☐ Heart problems
- ☐ Breathing problems
- ☐ Psychiatric problems
- ☐ Not applicable
- ☐ Other: ___________________

XIX. Type(s) of transport that you use most frequently:*

- ☐ Car
- ☐ Bus
- ☐ Boat
- ☐ Train
- ☐ Metro
- ☐ Bike
- ☐ Motorcycle
- ☐ By foot
- ☐ Taxi
- ☐ Other: ___________________

7.2. Perception about air quality

XX. How concerned are you about the following problems in your area?*

	1 – No Concern	2 – Minor Concern	3 – Moderate Concern	4 – Major concern
Traffic	☐	☐	☐	☐
Urban cleaning and hygiene	☐	☐	☐	☐
Waste Management	☐	☐	☐	☐
Sewage management	☐	☐	☐	☐
Air pollution	☐	☐	☐	☐
Noise	☐	☐	☐	☐
Over construction	☐	☐	☐	☐
Lack of green spaces	☐	☐	☐	☐

XXI. How do you rate the air quality at?*

	1 – Very poor	2 – Poor	3 – Acceptable	4 - Good	5-Very good
Portugal	◯	◯	◯	◯	◯
In your municipality	◯	◯	◯	◯	◯
In your neighbourhood	◯	◯	◯	◯	◯

XXII. According to your opinion, the air quality in your area of residence is:*

- ◯ Getting worse
- ◯ Maintaining
- ◯ Improving

XXIII. In your opinion, what are the pollutant sources that have the greatest impact on the air quality in your area of residence?*

- ◯ Cars
- ◯ Buses
- ◯ Trucks
- ◯ Motorcycles
- ◯ Boats
- ◯ Air traffic
- ◯ Fireplaces and other home heating devices with biomass burning
- ◯ Forest fires
- ◯ Burning of green wastes
- ◯ Agriculture
- ◯ Food making activities
- ◯ Solid waste (landfills, incinerators, dumps)
- ◯ Wastewater treatment
- ◯ Industry
- ◯ Energy production and distribution
- ◯ Construction
- ◯ Cigarette smoke
- ◯ Other: ___________________

XXIV. Which air pollutant(s) are you most concerned about?

____
____
____

XXV. Compared to outdoor air, indoor air quality in your home seems that it is:*

- ◯ Getting worse
- ◯ Maintaining
- ◯ Improving

XXVI. List the main sources that affect indoor air quality in your home: ______________________

7.3. Impacts of air quality

XXVII. Do you feel affected by air quality?*

- Yes
- No

XXVIII. If yes, in which way?

- Eye irritation
- Nose irritation
- Sneezing
- Dry throat or throat irritation
- Cough
- Difficulty in breathing
- Headaches
- Skin problems
- Allergies
- Feeling depressed
- Decreased outdoor visibility (fog)
- Unpleasant odour outside the house
- Unpleasant smell inside the house
- Particle deposition on balconies
- Degradation of material goods
- Concern for your health
- Concern for children's health
- Other: ___________________

XXIX. Did you identify the source of your malaise? *

- Yes
- No

XXX. If yes, please indicate the source of your malaise:

____

XXXI. Do you do changes in your life due to the degradation of air quality?

- Yes
- No

XXXII. If yes, which type of changes?

- To perform less outdoor activities
- To avoid certain time periods to perform outdoor activities
- To stay longer at home
- To avoid opening home windows

- To feel the need to wash hands and face more often
- To drink more water than usual
- To use purifiers at home
- To think about changing the area of residence
- To try to find out more about Air Quality
- To use a different type of transport
- Other: ____________________

7.4. Information about air quality

XXXIII. Do you feel informed about the air quality in your area of residence?*

	1	2	3	4	5	
Not at all	◯	◯	◯	◯	◯	Very much

XXXIV. Where do you get information about air quality? *

- TV
- Radio
- Newspapers and magazines
- Internet
- Electronic information boards
- Environmental groups
- Health services
- Town hall
- Parish Council
- Ministry of Environment
- QualAR
- Neighbours/Colleagues/Friends
- I can not access information
- Other: ____________________

Remark: QualAR is the Portuguese public and online database about air quality established by the Portuguese Environment Agency.

XXXV. Would you like to have more information about air quality?*

- Yes
- No

7.5. Control of Atmospheric Pollution

XXXVI. In your opinion, what is the degree of priority that do you give to the following air quality improvement measures?*

	1 (minimum)	2	3	4	5 (maximum)
Make stricter laws to reduce air pollutants	⬭	⬭	⬭	⬭	⬭
Increase enforcement to enforce existing laws	⬭	⬭	⬭	⬭	⬭
Improve public transport	⬭	⬭	⬭	⬭	⬭
Increase pedestrian networks	⬭	⬭	⬭	⬭	⬭
Increase bike paths	⬭	⬭	⬭	⬭	⬭
Increase car sharing	⬭	⬭	⬭	⬭	⬭
Increase tax benefits for electric cars	⬭	⬭	⬭	⬭	⬭
Improve the charging network for electric cars	⬭	⬭	⬭	⬭	⬭
Reduce the number of cars in cities	⬭	⬭	⬭	⬭	⬭
Reduce speed limits in cities	⬭	⬭	⬭	⬭	⬭
Limit parking in cities	⬭	⬭	⬭	⬭	⬭
Increase parking on the outskirts of cities	⬭	⬭	⬭	⬭	⬭

Create green spaces					
Carry out awareness-raising actions on air quality					
